# A Case of Right Alien Hand Syndrome Coexisting with Right-Sided Tactile Extinction

**DOI:** 10.3389/fnhum.2016.00105

**Published:** 2016-03-14

**Authors:** Michael Schaefer, Claudia Denke, Ivayla Apostolova, Hans-Jochen Heinze, Imke Galazky

**Affiliations:** ^1^Department of Neurology, Otto-von-Guericke University MagdeburgMagdeburg, Germany; ^2^Department of Anesthesiology and Intensive Care Medicine, Charité – Universitätsmedizin BerlinBerlin, Germany; ^3^Department of Radiology, Otto-von-Guericke University MagdeburgMagdeburg, Germany

**Keywords:** alien hand syndrome, corticobasal degeneration, tactile extinction, touch, vision

## Abstract

The alien hand syndrome (AHS) is a fascinating movement disorder. Patients with AHS experience one of their limbs as alien, which acts autonomously and performs meaningful movements without being guided by the intention of the patient. Here, we report a case of a 74-years old lady diagnosed with an atypical Parkinson syndrome by possible corticobasal degeneration. The patient stated that she could not control her right hand and that she felt like this hand had her own life. We tested the patient for ownership illusions of the hands and general tactile processing. Results revealed that when blindfolded, the patient recognized touch to her alien hand only if it was presented separated from touch to the other hand (bilateral asynchronous touch). Delivering touch synchronously to both the alien and the healthy hand resulted in failure of recognizing touch to the alien hand (bilateral synchronous touch). Thus, AHS here co-existed with right-sided tactile extinction and is one of only very few cases in which the alien hand was felt on the right side. We discuss the results in the light of recent research on AHS.

## Introduction

AHS is a bizarre and very rare neurological movement disorder first described by Goldstein (1908). Patients with AHS experience one of their limbs as alien, which acts autonomously and performs goal-directed movements that are not guided by the intention of the patient. For example, patients grasp for objects or touch their face without volition. Patients may even act aggressively against themselves. The patient is aware of the discrepancies between intentions and the actions of the hand. Often he or she tries to prevent the hand from moving by grasping it firmly with the other hand. Patients with AHS describe the experience of their alien limb as if someone else moves the alien hand (Goldstein, 1908). The patient reported by Goldstein (1908) complained that there must be an “evil spirit” in the hand. Consequently, AHS patients often call the alien limb in the third person. Nevertheless, patients are aware that the limb is still part of their body and do not deny the ownership of their alien limb when being asked (in contrast to cases of asomatognosia; Goldstein, 1908; Biran and Chatterjee, 2004; Fitzgerald et al., 2007).

AHS has been reported subsequent to lesions in various brain regions, for example supplementary motor area (SMA), anterior cingulate, corpus callosum, anterior prefrontal cortex, posterior parietal cortex, and thalamus (Goldberg et al., 1981; Martí-Fàbregas et al., 2000; Marey-Lopez et al., 2002; Scepkowski and Cronin-Golomb, 2003; Biran and Chatterjee, 2004; Assal et al., 2007; Fitzgerald et al., 2007; Brainin et al., 2008). According to the anatomical lesions and clinical features, different subtypes of AHS have been described (Bogen, 1993; Bundick and Spinella, 2000; Biran and Chatterjee, 2004). However, a clear anatomico-clinical correlation of the different clinical characteristics is still missing.

Hence, the neural mechanisms of this dissociation between will and action still remain unclear. Only very few studies tried to report neural correlates of these unwanted movements (Assal et al., 2007; Schaefer et al., 2010). Interestingly, recent studies tried to unravel the mechanisms of this peculiar movement disorder by making use of experiments that manipulate multisensory integration processes. For example, it has been demonstrated in healthy subjects that the bodily self can be easily disturbed by simple manipulations of multisensory integration (vision, touch, proprioception), resulting in ownership misattributions of seen or felt limbs (the so-called rubber hand illusion, RHI; Botvinick and Cohen, 1998; Armel and Ramachandran, 2003). In this illusion participants watched a life-sized rubber hand placed on a table in front of them while their own arm was hidden from view. Now the experimenter used two paintbrushes to touch both the rubber hand and the real hidden hand repeatedly in a synchronous way. After a while the participants felt the touch on the fake hand, suggesting the embodiment of the rubber hand. This ownership illusion disappeared or diminished when a small asynchrony was introduced between the stroking of the rubber and the real hand (Botvinick and Cohen, 1998). In our previous study, we tested a patient with AHS on a particular version of this illusion, the somatic rubber hand illusion (SRI), and found an interaction of experimentally induced body illusions (based on the manipulation of touch and proprioceptive information) with the alien hand. We observed strong movements of the alien hand within seconds whenever this body illusion started. The patient immediately used her healthy hand to stop the movements of the alien hand, because she felt very uncomfortable with these involuntary movements. Since we could provoke these movements of the alien hand reliable whenever we started the illusion, we got the impression that we could use this illusion to “wake up” the alien hand (Schaefer et al., 2013).

Another recent study used similar manipulations of visuotactile integration processes in order to influence the feeling of an alien hand. Romano et al. (2014) hypothesized that voluntary motor control could be improved by restoring the congruency between motor intentions and visual feedback. In order to test their hypothesis they employed a mirror box paradigm for a patient with AHS in the right hand. The mirror box consisted of an opaque box with a hole on the wall facing the patient, where she could introduce her hand, and a mirror featuring its parasagittal wall. The patient was asked to place her arms on the table, keeping the alien hand inside the mirror box and the other one outside the box, in front of the mirror. The patient now performed rhythmic tapping movements with both index fingers. Due to the mirrorbox the patient was able to see only her intact hand moving, resulting in a mirror reflection matching the image of the alien hand. The authors hypothesized that a training based on this mirror effect would increase motor control over the affected hand. In fact, the results demonstrated improved motor speed after the mirror box training and also a qualitative improvement of motor behavior of the alien hand. The authors explained these results by arguing that visual feedback provided by the mirror may have increased the sense of congruence between intention and sensory feedback (the visual information).

The present study examined visuo-tactile processing in a 74-year-old lady with right-handed AHS. The patient was diagnosed an atypical Parkinson syndrome by possible corticobasal degeneration. The aim of this study was to test if this patient may demonstrate similar interactions of visuo-tactile illusions with the alien hand as shown in our previous article (Schaefer et al., 2013). Given that we found a strong effect of the RHI on alien movements in our previous study, we hypothesized that the alien hand of our patient may interact with the illusion in a similar way. Thus, we assumed that a successful induction of a RHI in the patient would provoke alien hand movements. We tested two versions of the RHI, because the previous study showed effects only for the somatic version of the RHI. Furthermore, we conducted a third tactile experiment in order to proof general tactile processing in this patient.

## Case Report

The patient was recruited from the Department for Neurology of the Otto-von-Guericke University, Magdeburg, Germany. The study adhered to the Declaration of Helsinki and written informed consent was obtained from the patient.

The 74-year-old right-handed lady was diagnosed with Parkinson syndrome 4 years ago. She reported a progressive stiffness of her right hand and an irregular tremor later starting. Furthermore, she claimed that she could not control her right hand and that she felt like this hand had her own life. Sometimes she had difficulties to loosen the grip of this hand. Stiffness and tremor progressed to the left hand since 6 months and to the right leg since 2 months with subsequent gait disorder. Currently, the patient was not able to use her right hand for simple tasks.

Clinical examination revealed a strongly right-sided reduction of bodily movement (hypokinesia) with rigor and dystonia in the hand. Furthermore, we observed quick, involuntary muscle contractions of the right arm (myoklonus). The patient showed an uplifted right arm with reduced swing during gait. Reflexes were attenuated in the right arm. Detailed neuropsychological assessment revealed difficulties in motor planning (ideomotor apraxia), mirror movements, and tactile naming disorder. The patient reported episodes of tonic grasping.

**Figure 1:**
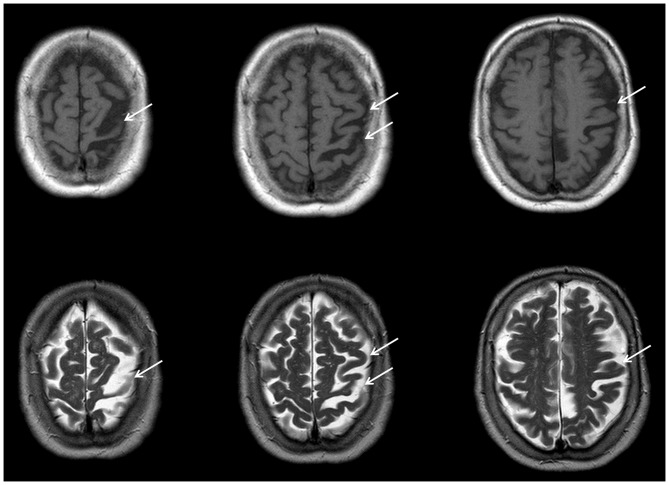
**Transaxial MR imgages of the patient.** T1-weighted (first row) and T2-weighted (second row), showing distinct atrophy of the left superior frontal and parietal cortices (arrows) involving predominantly the primary somatosensory (Brodmann areas 1, 2, 3) and primary motor cortex (Brodmann area 4). MR data were acquired with a 3 T Magnetom Trio Siemens scanner (3D-SPGR, TR = 24 ms, TE = 8 ms).

Structural MRI showed global atrophy with focus on the frontoparietal cortex and the left pre- and postcentral gyrus including primary somatosensory cortex (SI, Brodmann areas 1, 2, 3) and primary motor cortex (M1, Brodmann area 4; see Figure 1). Tracer studies (DAT-Scan and FDG-PET) revealed loss of presynaptic dopamine as well as an asymmetric hypometabolism of the left frontal, parietal and insular cortex as well as of the left caudate nucleus.

Based on the clinical presentation and imaging data we diagnosed an atypical Parkinson syndrome by possible corticobasal degeneration and AHS on the right side.

## Procedure

The patient participated in three tests. First, we examined the classic RHI in this patient. Second, we tested her with the SRI. Third, we examined general tactile detection in this patient.

For the classic RHI the participant is seated on a comfortable chair, with the arms placed on a table in front of him or her. A standing screen is used to hide the left (right) arm from the subject’s view. Then the experimenter places a life-sized rubber model of a left (right) hand on the top of the table (in the same perspective as the patient’ s real hand). The experimenter now uses two small paintbrushes to stroke both the rubber hand as well as the subject’s hidden hand in synchrony (or in asynchrony for control condition). Most of the participants (about 80%) soon develop a feeling of ownership for this rubber hand (Botvinick and Cohen, 1998). This is tested by a questionnaire the patient had to complete after each condition (left/right hand, synchronous, asynchronous). In this questionnaire the patient is asked to rate the degree of agreement with five statements extracted from the studies of Botvinick and Cohen (1998) and Ehrsson et al. (2004). Thus, the patient is asked if during the experiment he felt as if the rubber hand was his own hand, if he felt the touch of the paintbrush in the location where he saw the rubber hand touched, if his own hand felt artificial, if he felt his own hand moving, and if he had the feeling to have more than one left (right) hand. The first and second statement indicate the occurrence of the illusion, the other statements were control questions.

In contrast to the previous illusion the SRI needs the patient to be blindfolded. A life-sized rubber model of a hand is placed on the table between the participant’s hands. Now the experimenter moves the patient’s left index finger so that it touches the right rubber hand on the knuckle of the index finger. Simultaneously the experimenter also touches the knuckle of the index finger of the patient’s right hand in a synchronous way. In a control condition the experimenter touches the hands in an asynchronous manner. If touching is done in a synchronous way, participants feel a strong illusion that they were touching their own hand (instead of the rubber hand; Ehrsson et al., 2005). The occurrence of the illusion is again tested by a questionnaire according to the study by Ehrsson et al. (2005). The participant is asked if he felt as if he was touching his right hand with his left index finger (and* vice versa*, respectively), if he felt more than one left (right) hand, if he had the feeling that his own hand felt larger than normal, if he had the feeling that the own hand was moving, and if he had the impression of not feeling the own hand anymore. The first statement indicates the occurrence of the illusion, the other statements were control questions. For both experiments, the patient had to indicate his responses on a seven-point scale ranging from “completely disagree” (−3) to “completely agree” (+3). For further methodological details for both experiments see Schaefer et al. (2013).

Last, we applied a tactile detection task (e.g., Gainotti et al., 1975; Schwartz et al., 1977). Here the patient received touch with the experimenter’s finger to the subjects’s hands and arm. The examination included 20 trials of asynchronous (single touch) or simultaneous light touches to the palm of the hands, in a random order. Synchronous touch represented touch applied to roughly the same portions on both sides of the body. The blindfolded patient was asked to respond if she felt touch on both hands or only one hand (or arms, respectively). Further conditions (always 20 trials) included stronger touch to the hands and different locations of the touch (thumb, index finger, upper arm; synchronous touch engaged always the analog body part of the other side of the body). In addition, conditions involved light touch to the palm of hands with eyes open and light touch to the palm of the hand with a stick and paintbrush, respectively. When leaving the eyes open, we arranged that the patient was still not able to see the actual stimulation (by using a paperboard; non-informative vision). Thus, the patient was able to see her body and most portions of the arm and the hand, but not the actual stimulation. The trials were applied in a random order.

## Results

For both classic RHI and SRI the patient failed to feel any illusions that are known for the majority of healthy subjects. All questions of the questionnaires were completely refuted for all runs and both experiments (−3 on the seven-point scale ranging from “completely disagree” (−3) to “completely agree” (+3)). Thus, the patient responded to not feel any illusion at all (regardless of the hands; see Table 1).

**Table 1:** **Results of rubber hand illusion (RHI) and somatic rubber hand illusion (SRI)**.

	Alien hand	Healthy hand
**RHI**
I felt as if the rubber hand was my own hand	−3	−3
I felt the touch of the paintbrush in the location where I saw the rubber hand touched	−3	−3
I felt my own hand artificial	−3	−3
I felt my own hand moving	−3	−3
I had the feeling to have more than one left (right) hand	−3	−3
**SHI**
I felt as if I was touching my right (left) hand with my left (right) index finger	−3	−3
I felt more than one left (right) hand	−3	−3
I had the feeling that my own hand felt larger than normal	−3	−3
I had the feeling that the own hand was moving	−3	−3
I had the impression of not feeling the own hand anymore	−3	−3

*Patient indicated the response on a seven-point scale ranging from “completely disagree” (−3) to “completely agree” (+3). Underlined questions indicate the occurrence of the illusion, the other statements are control questions*.

In the third test, we found that when touch applied to both the left healthy hand and the right alien hand in an *asynchronous* way, the patient told us to feel this touch clearly on the alien hand as well as on the healthy hand (in 100% of all trials). Furthermore, the patient stated that she felt this touch on both hands in the same way and strength. In contrast, when touching the healthy hand and the alien hand in *synchrony*, the patient claimed to feel touch on the healthy, but nothing at all on the alien hand (again, in 100% of all trials). This result could reliably be replicated.

The patient failed to detect synchronous touch on the alien hand even if we increased the force of touching. Touching the hands with a small stick or a soft paintbrush revealed the same result. Furthermore, she still did not feel any synchronous touch if we changed the site of touching on the hand (e.g., different fingers, palm of the hand). However, when applying synchronous touch to the upper arms, she was able to detect this stimulation. In addition, allowing the patient to see the stimulation (non-informative vision) revealed no failure in the detection of synchronous touch (see Table 2 for detailed results).

**Table 2:** **Results of behavioral testing when applying light touch with index finger of the experimenter**.

	Alien hand	Healthy hand
Synchronous touch to the palm of both hands	No touch felt	Touch felt
Asynchronous touch to the palm of both hands	Touch felt	Touch felt
Synchronous touch to the D1 of both hands	No touch felt	Touch felt
Asynchronous touch to the D1 of both hands	Touch felt	Touch felt
Synchronous touch to the D2 of both hands	No touch felt	Touch felt
Asynchronous touch to the D2 of both hands	Touch felt	Touch felt
Synchronous touch to the upper arm	Touch felt	Touch felt
Asynchronous touch to the upper arm	Touch felt	Touch felt

*Results were identical when using a stick or a paintbrush*.

## Discussion

AHS is a very rare movement disorder characterized by involuntary and unwilled purposeful movements usually of the left hand. Here, we present a patient with right-handed AHS coexisting with right-sided tactile extinction.

Several studies describe single cases of AHS subsequent to different anatomical lesions in the patients. However, AHS is usually described as a consequence of right hemisphere, resulting in a left-handed alien hand. Only few cases report right-handed AHS. For example, Della Sala et al. (1991) describe a right-sided alien hand in a patient with a bilateral frontal vascular lesion and damage of the corpus callosum. McNabb et al. (1988) report on a patient who had right-handed AHS subsequent infarction of the left superior and medial frontal and parietal cortex and of the corpus callosum. More recently, McBride et al. (2013) describe an alien hand behavior of the right hand in a patient with corticobasal syndrome. Our own recent study reports a right-handed alien limb after acute ischemic stroke (arteriosclerosis of the left internal carotid artery; Schaefer et al., 2013). Romano et al. (2014) describe a patient with right AHS subsequent an intracerebral hemorrhage in the left fronto-parietal region. The authors argue that the right-handed AHS cannot be explained by atypical hemispheric lateralization, because their patient expressed full right-handedness.

In contrast to our previous patient we were unable to elicit ownership illusions such as the classic RHI or the SRI. Hence, we could not detect any interactions of the alien limb with possible tactile illusions. Although many healthy participants fail to experience the RHIs, too, the lack of illusion in our patient may be explained by the result of our third experiment. Thus, we found that our patient detected touch to her right alien hand only if it was presented separated from touch to the other hand (asynchronous touch). Delivering touch synchronously to both the alien and the healthy hand resulted in failure of recognizing touch to the alien hand. Delivering touch to the healthy hand revealed correct detection of this simulation regardless if we touched the alien hand synchronously or asynchronously. These tactile impairments have been described as tactile extinction (e.g., Gainotti et al., 1975; Schwartz et al., 1977). Patients show extinction behavior when they report to a stimulus in isolation but are unable to respond to the same stimulus presented simultaneously with another stimulus on the other side. Extinction phenomena are known not only for the tactile modality. For example, visual extinction (pseudohemianophobia) has been described as the inability to perceive two simultaneous stimuli in each visual field. Auditory extinction is defined as the failure to hear simultaneous stimuli on the left and right sides. It has been hypothesized that extinction is related to neglect and may be associated with higher level of input processing (Brozzoli et al., 2006). Similar to AHS, tactile extinction phenomena are more commonly associated with right than with left hemisphere lesions.

The patient in the current study is one of only very few cases of AHS coexisting with tactile distinction. Lin et al. (2007) describe AHS and left handed tactile extinction in a patient with mixed frontal and callosal type. Their patient was characterized by ischemic stroke of the left and right corpus callosum. To our knowledge, so far there are no reports on patients with corticobasal degeneration and both AHS and tactile sensory extinction.

Interestingly, our patient showed tactile extinction only when closing the eyes. Non-informative vision resulted in correct recognition of touch. For the perception of our own body visual and tactile senses are particularly important. Information of both modalities has to be integrated to produce a coherent internal representation. Recent studies suggest cross-modal links between vision and somatosensation at an early stage of sensory processing (e.g., Driver and Spence, 1998). In addition, recent studies have demonstrated behaviorally that viewing the stimulated body part can enhance tactile detection and discrimination ability at the stimulated site. Kennett et al. (2001) measured tactile two-point discrimination thresholds on the forearm while manipulating the visibility of the arm. They reported an improved tactile performance when the participants could see the arm, but no improvement when a neutral object was shown at the arm’s location. This visual-tactile enhancement seems to last several seconds up to minutes (Taylor-Clarke et al., 2002, 2004; Ro et al., 2004). Another study reports that viewing the arm could speed up reactions to an invisible tactile stimulus on the arm (Tipper et al., 1998, 2001). Hence, cross-modal interactions may enhanced tactile abilities in the present patient, who was able to detect simultaneous touch on both hands when opening the eyes (without seeing the stimulation directly). Thus, one may speculate that our patient may benefit from multisensory trainings (e.g., Serino et al., 2007; for stroke patients).

However, although alien hand and tactile extinction in our patient affected the same side, we feel unable to disentangle if these symptoms are related. Due to the multiple lesions caused by corticobasal degeneration (here, for example, lesions in pre- or postcentral areas), tactile extinction may be independent from the AHS in this patient. Future studies are needed testing other AHS patients for tactile extinction phenomena in order to understand possible relationships between AHS and extinction. Moreover, although we did not find any interactions with the alien limb for this patient (in contrast to our previous study), we believe that future studies should further try to employ approaches from cognitive neuroscience in order to help understanding this peculiar movement disorder, which neural underpinnings are still unclear, and for which we still have no established treatments.

## Author Contributions

Designed the experiment: MS and IG. Wrote the manuscript: MS and CD. Imaging: IA. Supplied material: H-JH.

## Conflict of Interest Statement

The authors declare that the research was conducted in the absence of any commercial or financial relationships that could be construed as a potential conflict of interest.
